# The Impact of the Interactive Floor Device and Aerobic Training on Executive Functions in Children

**DOI:** 10.3390/children11121489

**Published:** 2024-12-06

**Authors:** Krystyna Rymarczyk, Iwona Makowska, Sylwia Hyniewska

**Affiliations:** 1 Department of Biological Psychology, Faculty of Psychology, SWPS University in Warsaw, 03-815 Warsaw, Poland; imakowska@swps.edu.pl; 2 Independent Researcher, 03-815 Warsaw, Poland; sylwia.hyniewska@gmail.com

**Keywords:** physical activity, development, executive functions, young children, interactive floor

## Abstract

Background/Objectives: Considering the importance of physical activity on the development of cognitive functions in children, the aim of this study was to assess the effects of a ten-week training program using the Interactive Floor device (© Funtronic), i.e., a kinesthetic educational game, and aerobic activity training on executive functions in 9-year-old children. Given current knowledge of the advantages of gamification and on-task switching, stronger improvement was expected for the Interactive Floor device than aerobic exercise activities. Methods: Sixty-four children (29 boys/35 girls) were randomly assigned to the Interactive Floor (n = 22), Aerobic Training (n = 22), or Control groups (n = 20). The participants had their cognitive abilities assessed twice (pre- and post-intervention) using computer tests from the Vienna Test System (VTS) and subtests from the Wechsler Intelligence Scale for Children^®^ Fifth Edition (WISC^®^-V). From VTS, the Stroop Test was used to measure inhibition and attentional control, while the Corsi Block test assessed visuospatial short-term working memory. To assess auditory working memory, the Digit Span subtest from the WISC^®^-V was applied. Additionally, fluid intelligence was estimated using Raven’s Progressive Matrices. Results: Repeated-measures mixed ANOVA and post hoc tests with Bonferroni correction for multiple comparisons showed that all intervention program groups improved in terms of intelligence and non-verbal abstract reasoning. The second significant finding in this study was that especially children from the Interactive Floor group developed their executive functions, i.e., inhibition and attentional control as well as their spatial short-term memory capacity. Conclusions: The results suggest that a combination of both physical exercise and cognitive games in the Interactive Floor group resulted in greater improvement in cognitive abilities in children than aerobic exercise or physical education lessons. It seems that a multidisciplinary approach combining physical and cognitive stimulation effectively promotes child development. Future programs aiming to improve cognitive skills in children should consider incorporating interactive and engaging activities that stimulate both the body and the mind.

## 1. Introduction

There is a growing concern about decreased physical activity among children, especially compared to earlier generations [1,2,3]. Presently, children are adopting more sedentary routines, often engaging with computers, tablets, and television [4,5,6,7]. Consequently, they are forsaking the typical childhood games relying on gross motor skills and the customary physical activities integral to their developmental phases [8,9]. This deficiency in physical engagement gives rise to a range of health issues, encompassing posture irregularities [10], somatic ailments [11], excessive weight, and obesity [12,13]. The holistic impact of sports engagement extends to motor, cardiovascular, respiratory, hormonal, immunological, and nervous systems [14,15]. These dynamics collectively trigger the maturation of cerebral motor areas, subsequently influencing motor development [16] and accelerating nerve impulse conduction [17]. Moreover, physical activity induces the release of neurohormones, pivotal agents generated by hypothalamic neurons and conveyed through blood or cerebrospinal fluid, crucially modulating the excitability of synaptic neurons [18]. Consequently, the importance of regular physical activity for the comprehensive growth of both the body and the mind cannot be emphasized enough.

Empirical evidence progressively substantiates the link between insufficient physical activity and indicators of mental well-being [19,20,21]. Specifically, it was shown that non-regular physical activity is associated with higher levels of depression [22], stress [23], and overall psychological distress [24]. Moreover, adolescents grappling with weight issues who are devoid of sports involvement are predisposed to engaging in risky behaviors, including self-harm [25], as well as alcohol and illicit drug dependency [26]. On the other hand, higher levels of physical activity are correlated with enhanced psychological well-being, which encompasses factors like a positive self-image, life satisfaction, happiness, and overall psychological wellness [19]. These findings underscore the important role of physical activity in promoting mental health and emotional resilience in children [27,28,29].

While the correlation between physical activity and both physical and mental health is well established [30,31], there remains a need for more extensive investigation into the link between physical activity and cognitive functioning. The research [28,32] underscores that optimal cognitive functioning among preadolescents necessitates more than just a satisfactory intelligence quotient (IQ); it also hinges upon the robust development of executive functions encompassing inhibition, working memory, and cognitive flexibility [33]. Notably, these executive functions are nurtured through engagement in sporting activities in both children [34,35,36] and adults [37,38,39]. The empirical studies primarily focus on inhibition playing a central role within the broader framework of executive function [40,41]. Inhibition allows children to control their attention, behavior, thoughts, and emotions, enabling them to take the most appropriate action in a given situation. It involves overriding a strong internal predisposition or blocking habitual or unsuitable actions. Recent meta-analyses have consistently demonstrated that regular physical activity positively correlates with improvements in inhibition [42] and attentional control [35] in children. Interestingly, even a 50 min sports-based physical education class performed at moderate intensity has been shown to improve reaction time in the Stroop test in 10-year-old children [43]. As the authors of the cited study suggest, a possible explanation for the observed improvement might be the increase in cerebral oxygenation. It has been demonstrated that moderate-intensity exercise enhances cognitive performance and boosts activation in the prefrontal cortex [44,45], a brain region crucial for executive functions such as inhibitory control and attentional focus. On the other hand, it was shown that children involved in long-lasting physical training (9–13 weeks of aerobic games) showed decreased frontal activation associated with better cognitive control [46,47]. Together, these findings suggest that physical activity plays a key role in improving brain functioning, particularly in the frontal cortex, and that as the brain matures, it becomes more efficient, requiring less activation to achieve superior cognitive performance [48].

Working memory refers to the short-term storage and manipulation of information necessary for effective action [49]. It allows children to interrelate and reorganize pieces of information, translate instructions, and incorporate new information into their action plans. This function is essential for complex cognitive tasks, such as problem-solving and understanding instructions. A recent meta-analysis by Vasilopoulos et al. [50], encompassing data from 92 studies, reveals that physical activity significantly enhances cognitive functions in children, particularly in the domain of working memory. The analysis covered a wide range of physical activities, from dance to aerobic exercise, and found that these activities notably improve working memory in children aged 5 to 12 years. Indeed, in the study of 10–11-year-old children, positive correlations were found between physical activity level and digital span, as well as the Corsi block test [51]. A similar improvement was observed in children who trained in karate [52], tennis, or football [53]. These findings underscore the broad cognitive benefits of physical activity across various types of exercise. It should also be emphasized that children who are more physically fit tend to have larger volumes of hippocampus, which play a critical role in memory formation, as well as larger volumes of basal ganglia associated with cognitive control [54]. To conclude, well-rounded cognitive development necessitates the development of executive functions, which are all encouraged during engagement in sports activities [28]. However, there is ongoing research and debate concerning which type and intensity of physical activity can influence various aspects of cognition [22,55]. The established consensus emphasizes that acute exercise triggers an immediate neurochemical response that potentially heightens cognitive performance [56]. Aerobic exercise training yields targeted, rather than all-encompassing, effects on children’s cognitive capabilities with a discernible enhancement in children’s performance in executive function [34]. Some authors contrast aerobic exercises with complex physical disciplines regarding the use and development of executive functions. Thus, martial arts are known for a more all-encompassing development beyond fostering executive functions and the generalizability of the acquired new skills to new domains [34,57,58]. Indeed, it seems that exercise conducted within a cognitively engaging context exhibits a more pronounced impact on the brain [59]. For example, it has been shown that challenging but gamified activities that are physically and cognitively engaging simultaneously and naturally require various executive functions can be more effective on all fronts than sedentary–cognitive exercises targeting one executive function at a time [60].

Contemporary children grew up at the advent of digital technology. The realm of digital engagement, encompassing computer games, social media, and virtual reality, exerts a substantial influence on children who are introduced to these technologies at tender ages. These technologies have manifested their impact in domains ranging from studying and recreational gaming to social interaction, and consequently, incorporating digital games into educational toolboxes is not surprising [61,62]. Indeed, exercise-based interventions can be led through very engaging interactive games. Such games providing both entertainment and physical exertion called “exergames”, facilitate a dynamic, full-body gaming experience [60,63]. Research has found that exergaming can lead to improvements in physical fitness, motor skills, and academic performance in children [64] and even have some positive effects on self-concept, motivation, and social well-being [65].

The latest meta-analysis [66], which involved 11 randomized experimental studies (n = 508 children), demonstrated that exergaming positively affects children’s inhibitory control, working memory, and cognitive flexibility. Furthermore, the findings suggest that exergaming may enhance children’s executive functions more effectively than traditional non-exercise video games or even single sessions of aerobic exercise [67,68]. For example, one of the studies [68] demonstrated that 4–5-year-old children who participated in an 8-week exergaming training program showed significant improvements in their results on the Dimensional Change Card Sort (DCCS) Test. Additionally, these children displayed significantly greater increases in executive functions compared to those who engaged in traditional physical training activities, such as tag games, locomotion activities, or soccer. This suggests that exergaming, cognitively engaging physical activities, may be particularly effective in enhancing executive functions in young children, potentially offering advantages over more conventional forms of physical activity.

Considering the importance of physical activity on the development of cognitive functions of children as well as children’s interest in computer games, the objectives of this study were to assess how the use of the Interactive Floor, a kinesthetic educational game, affects executive functions in 9-year-old children. Since most studies focus on the impact of aerobics on executive functions in children, we decided to compare the impact of both activities, aerobics and exergaming. Additionally, for the control group, children without any extracurricular sports activities were selected. It was hypothesized that children participating in the Interactive Floor training would demonstrate superior performance compared to the control group, particularly in executive functions such as inhibitory control, attentional regulation, and visuospatial working memory capacity. Likewise, it was anticipated that children undergoing aerobic training would also show improvements in these executive domains following the intervention, although the magnitude and nature of these changes might differ from those observed in the Interactive Floor training group. It was also expected that children in the control group would exhibit some age-related improvements in executive functions, reflecting the natural developmental progression of cognitive abilities over time.

## 2. Materials and Methods

### 2.1. Participants

All students from classes at level 3 of education from the local elementary school Strumienie, located in Józefów in Poland, were invited to take part in the study (N = 122). A total of 64 participants (mean age = 9.16, SD = 0.59, range = 9–11 years, 29 boys (45.31%) and 35 girls (54.69%) took part in the study. None of the children had a history of developmental, neurological, or psychiatric disorders and an absence of uncorrected sensory alterations. Children were attributed in a randomized order to one of the intervention groups, i.e., Interactive Floor training (IF, n = 22) or Aerobic training (AT, n = 22), and to the Control group with unmoderated free play (CON, n = 20).

### 2.2. Overall Design and Procedure

All children participated in the program for 10 consecutive weeks. The sessions were held in a school gymnasium 5 times a week for 45 min periods after school lessons. The participants were assessed twice (pre- and post-intervention) for cognitive ability using the computer tests from the Vienna Test System (VTS), subtest from the Wechsler Intelligence Scale for Children^®^ Fifth Edition (WISC^®^-V), and Raven’s Progressive Matrices. The first measurements of cognitive skills were carried out within 10 days before the training program. The post-tests were performed within 2 weeks after the last session. To carry out all tests, children were placed in a computer room and were tested individually by a child psychologist. Cognitive testing for each child lasted approximately 40 min. The study was conducted in accordance with the Declaration of Helsinki and approved by the Ethics Committee of University SWPS in Warsaw (protocol code no. 40/2024) for studies involving humans. The written informed consent was provided by the children’s legal guardian, as well as all children gave their consent to participate in the study.

### 2.3. Interactive Floor Training

The Interactive Floor (© Funtronic) platform that was used consisted of a projector, motion sensors, speakers, and a computer in one device (https://funtronic.eu/en; accessed on 20 November 2024). Each session began with a set of warm-up exercises which required intensive movement and raising the children’s heart rate as well as familiarizing them with the use of the Interactive Floor. The warm-up was followed by exercises aimed at acquiring specific knowledge or developing specific skills such as working memory, inhibition, and reasoning. The sessions always ended with exercises focusing on social skills, relaxation, or fun. Examples of games in each part are presented in Table 1. In this training, all sessions were conducted by a moderator trained and qualified to run the Interactive Floor games.

### 2.4. Aerobic Training

Each session began with a warm-up (10 min) with stretching and aerobic walking followed by progressive aerobic, muscular, and motor skills exercises (25 min) like jumping or tag and ball games, aerobic dance steps with an appropriate music background to the movements as well as quick running in place, 15 m shuttle run and obstacle course. The session ended with a cool-down phase (10 min) involving tape exercises or a hula hoop. In order to maintain the motivation of the participants, different exercises from moderate to vigorous intensity were applied. All sessions were conducted by a physical activity specialist—both the education teacher and personal trainer.

### 2.5. Cognitive Tests Measures

The tests were the Stroop Test and the Corsi block test from the Vienna Test System—VTS. From WISC^®^-V, Digit Span was applied. Table 2 presents a brief description of the used tests. The order of administration of the tests was defined in a systematic and pseudo-random manner, i.e., all subjects began by completing the Raven’s Progressive Matrices then one-half of the respondents took computer tests, while the other half started with the WISC^®^-V subtest.

### 2.6. Statistical Analyses

The analyses were conducted using IBM SPSS Statistics (version 29.0.0.0 (241)). To investigate the impact of the 10-week training programs on cognitive functions, a series of repeated-measures mixed ANOVA with fixed factors, i.e., Group (IF, AT, and CON) and Time (pre and post-test) as well as post hoc tests with Bonferroni correction for multiple comparisons were used for each dependent measures. To assess whether there were significant changes in cognitive test results from before to after the intervention within each specific group, separate paired *t*-tests were performed. Because the ANOVA showed no significant main or interaction effects for the participant’s gender, this factor was not considered further, and data obtained from the girls and boys were combined in each test. For all analyses, a statistically significant level was defined as *p* < 0.05. Effect sizes of repetition factors were expressed using partial eta squared (ηp 2).

## 3. Results

### 3.1. Stroop Test

**Figure 1:**
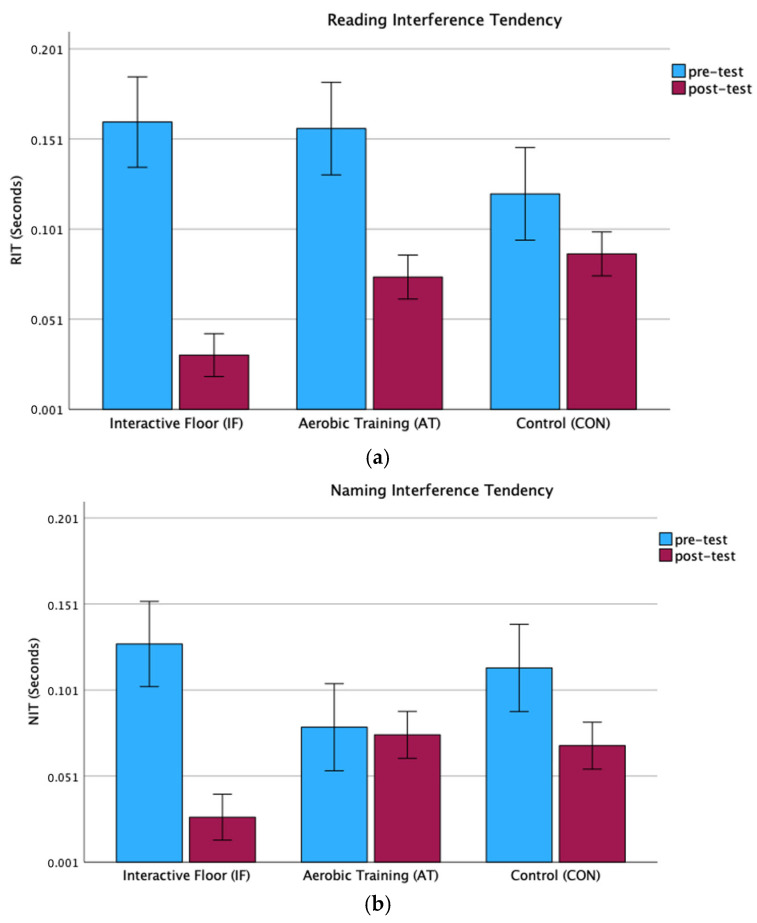
(**a**) Reading interference tendency (RIT) in the Interactive Floor, Aerobic training, and Control groups. (**b**) Naming interference tendency (NIT) in the Interactive Floor, Aerobic training, and Control groups.

Results of the repeated-measures mixed ANOVA conducted for two conditions of Stroop performance, i.e., Reading Interference Tendency (RIT) and Naming Interference Tendency (NIT) revealed that there was significant main effect for Time (F(1, 61) = 26,948, *p* < 0.001, ηp 2 = 0.030) showing decreased reaction time (RT) post-intervention with an average of 0.126 (SD = 0.012) before and 0.061 (SD = 0.05) after training, irrespective of group (Table 3). We also obtained a statistically significant effect of the Stroop condition (F(1, 61) = 4676, *p* = 0.035, ηp 2 = 0.071) where, for the NIT condition, the subjects received less time (mean = 0.082, SD = 0.009) than for the RIT condition (mean = 0.105, SD = 0.008). There was no effect of Group (*p* > 0.1); however, analysis reveals significant interaction Time by Group (F(2, 61) = 3814, *p* = 0.028, ηp 2 = 0.11). To clarify the interaction of Time (pre- and post-interventions) and Group, separate mixed ANOVAs revealed the effect of Group for Reading Interference Tendency (F(2, 61) = 6001, *p* = 0.004, ηp 2 = 0.016) and Naming Interference Tendency (F(2, 61) = 3741, *p* = 0.029, ηp 2 = 0.01), which are measures in the second time (post-test). Post hoc analysis with Bonferroni has shown a statistically significant difference between the IF and AT group (*p* = 0.041) and between IF and CON (*p* = 0.005) for the RIT condition. For the NIT condition, similar differences were obtained, i.e., between the IF and AT group (*p* = 0.044) and between IF and CON (*p* = 0.049). There was no significant difference for both condition measures in the second time between the Aerobic training and the Control group (Figure 1a,b). Separate paired *t*-tests for the groups (IF, AT, and CON) have shown that the RT for both Stroop conditions decreased in the IFT group (*p* < 0.001 for RIT; *p* = 0.005 for NIT), in the AT group for RIT (*p* = 0.012), as well as in the CON group for NIT (*p* = 0.034).

### 3.2. Corsi Block

**Figure 2:**
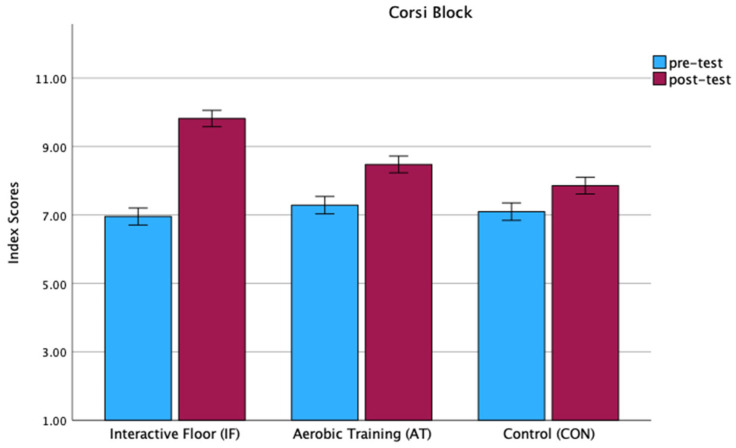
Corsi Block index scores in Interactive Floor, Aerobic training, and Control groups.

A repeated-measures ANOVA revealed a significant main effect of Group on Corsi Block index scores (F(2, 61) = 5.98, *p* = 0.004, η^2^p = 0.164) with an average of 8.386 (SD = 0.184) for Interactive Floor, 7.881 (SD = 0.189); for Aerobic Training, 7.476 (SD = 0.189); and for the Control group (Table 4). We also obtained a significant effect of Time (F(1, 61) = 73.06, *p* < 0.001, η^2^_p = 0.545), suggesting higher scores in the second measure (mean = 8.717, SD = 0.141) compared to the first one (mean = 7.112, SD = 0.146). The interaction Time × Group (F(2, 61) = 11.82, < 0.001, η^2^_p = 0.279) also reached statistical significance. Post hoc analysis with Bonferroni correction showed that, during the pre-test, there were no significant differences between the groups Interactive Floor vs. Aerobic (*p* = 1.000), Interactive Floor vs. Control (*p* = 1.000), and Aerobic vs. Control (*p* = 1.000) (Figure 2). However, after the intervention (post-test), a significant difference emerged between the Interactive Floor group and the Aerobic group (*p* < 0.001), as well as the Interactive Floor group and the Control group (*p* < 0.001). No significant difference was found between the Aerobic group and the Control group (*p* = 0.238). Separate paired *t*-tests for the groups (IFT, AT, and CON) showed that Corsi Block index scores increased in the IF group (*p* < 0.001), in the AT group (*p* < 0.001), and in the CON group (*p* < 0.02).

### 3.3. Digit Span

A repeated-measures ANOVA revealed a significant main effect of Time on Digital Span index scores (F(1, 61) = 16.66, *p* < 0.001, η^2^p = 0.214), suggesting that all children scored higher in the second measure (mean = 15.750, SD = 0. 338) compared to the first one (mean = 14.862, SD = 0. 377). The main effect of the Group (*p* = 0.716) and the interaction Time by the Group (*p* = 0.876) were not statistically significant (Table 5).

### 3.4. Raven’s Progressive Matrices

A repeated-measures ANOVA revealed a significant main effect of Time on Raven’s results (F(1, 61) = 42.423, *p* < 0.001, η^2^_p = 0.410), suggesting that all children scored higher in the second measure (mean = 94.841, SD = 0. 878) compared to the first one (mean = 87.066, SD = 1. 843). The main effect of the Group (*p* = 0.787) and the interaction Time by the Group (*p* = 0.125) were not statistically significant (Table 6).

## 4. Discussion

Given the rising concern over reduced physical activity among children due to increased use of technology such as smartphones and tablets [4,6], exergaming, which blends physical exertion with cognitive challenges, might not only match but potentially exceed the benefits of traditional physical activities in promoting physical [64] and cognitive development [66]. This study aims to provide insights into whether the combination of physical and cognitive exercise through the Interactive Floor can offer enhanced benefits for children’s executive functions compared to aerobic activities and standard physical education.

The primary findings of the present study were that in children from all study groups, an improvement in cognitive performance was observed after intervention programs. Particularly, these improvements (assessed through pre- and post-training measures) were found in both conditions of the Stroop Test, in the Block Corsi test, as well as in Raven’s Progressive Matrices. Our results in the scope of physical activity agree with those of previous studies, in which aerobic training [35,69,70], physical exercises with cognitive loads [71], exergaming [66,67] as well as physical education sessions in school [72,73] have improved children’s cognitive abilities.

Importantly, we found that, specifically, subjects in the Interactive Floor group outperformed those in the Aerobic and Control groups on the Stroop Test in both reading and naming interference conditions. The shorter reaction times demonstrate their better ability to inhibit automatic processes (like reading the word instead of naming the ink color). They were also better at overcoming interference when faced with conflicting stimuli (e.g., mismatched words and ink color). Our results are in line with the study of 8–12-year-old children, which showed that a 22-week program with cognitively engaging physical activities resulted in an enhanced inhibition measured using the Stroop test [74]. The findings of Schmidt et al. [75] also confirmed that a program with a high level of physical exertion and high cognitive engagement (team games group) had a selective effect on executive functions. After a 6-week intervention, the children from the team games group improved their performance in the inhibition and switching (Flanker task), but no training effect was found for a matched group focusing only on Aerobic exercises. These findings, together with ours, are consistent with the results of a meta-analysis review that found that exposure to cognitively demanding physical activity has a greater beneficial effect on neurocognitive functioning compared with aerobic exercise alone [35,76,77]. The obtained results also support the cognitive stimulation hypothesis [78,79,80], which states that interventions that include both high levels of cognitive engagement and physical effort have stronger effects on executive functions than physically demanding physical activity with low levels of cognitive engagement. Regarding brain mechanisms, it is suggested that the increased ability to control/inhibit and switch tasks that are observed after physical training involving cognitive challenges is mainly due to improved hemodynamics in the prefrontal cortex [81].

Despite our initial assumption, we did not observe a significant difference in reaction times in the reading and naming interference conditions between the Aerobic training and the Control groups. However, it is noteworthy that reaction times decreased significantly in both groups after the intervention. One possible explanation for this outcome is that the children in the Control group participated in regular physical education lessons twice a week. This consistent exposure to physical activity, even without specific aerobic training, may have contributed to the improvement in reaction time. For example, Kolovelonis et al. [72] have shown that eight 45 min physical education sessions in 9-year-old pupils, delivered over a period of four weeks, improved from pre- to post-intervention reaction times in the Stroop test. Moreover, a study carried out by Layne et al. [82] found that even a 10 min break with the FitNexx active video game resulted in an improvement in the executive functions in 8- and 9-year-old children. These findings align with recent reviews [73], highlighting that incorporating physical activity, even as small as including breaks from sitting by the desk in the classroom setting, is a highly effective strategy for enhancing cognitive and academic development in children.

The development of working memory skills is closely linked to children’s health and behavioral performance [83,84]. Li and Geary et al. [85] suggested that especially improvements in visuospatial memory in early childhood, i.e., between first and fifth grades, are linked to better mathematical achievement in a later period, even when other variables like prior academic performance, intelligence, and additional factors are accounted for. In our study, two indicators—verbal and spatial memory—were selected to assess the subjects’ cognitive capacity. The results indicated that, following the intervention period, spatial memory, as measured by the Corsi Block test, significantly improved in the Interactive Floor group compared to the Aerobic and Control groups. However, some expansion of working memory capacity was also seen in the Aerobic group after the intervention. The improvement in spatial, but not verbal, working memory might be attributed to the visuospatial characteristics of the exercises conducted on the Interactive Floor. They included activities that enhance coordination, e.g., jumping on one leg, balance (e.g., tightrope walking), spatial orientation (e.g., looking for chestnuts under the leaves), as well as memory (e.g., memorizing the number and location of ducks in a lake). Previous studies indicated that especially long-term training is related to higher working memory capacity [86] and spatial ability [87]. In a study carried out by Moreau et al. [88], 318 children aged 7–13 years were randomly assigned to a high-intensity training or an active control group. The children who took part in the high-intensity training outperformed their peers in the Corsi Bloc test. Moreau et al. [88] suggested that short but intense bursts of exercise can greatly impact brain functioning. Specifically, it is noted that exercise increases blood flow to the brain, which in turn leads to a rise in brain-derived neurotrophic factor (BDNF) [89], leading to neurogenesis as well as synaptogenesis, dendrite growth, and axonal branching [90]. Finally, enhanced performance on various learning tasks, as well as encouragement of long-term memory in the hippocampus, might be observed [54]. In conclusion, physical activity is one of the most potent and wide-ranging methods currently available to enhance cognitive function in children [88,91]. This is particularly important because, in the classroom, both cognitive control and working memory are closely associated with effective learning and overall achievement [92]. For example, it has been shown that the capacity of working memory correlates with performance in mathematics tasks [93] or reading accuracy [94].

When examining the existing scientific literature, there are relatively few studies addressing the relationship between physical activity and fluid intelligence in children. Moreover, the results are often contradictory. For example, findings of correlational studies [95,96,97] suggest that physically active children tend to have higher IQ levels compared to their inactive peers. In contrast, a study by Fochesatto et al. [98] involving 317 schoolchildren found no association between cardiorespiratory and muscular fitness and fluid intelligence. It was also shown [99] that additional physical activity as part of the curriculum during the school day did not provide benefits for fluid intelligence in children. The results of our study revealed that after interventions, children from all tested groups demonstrated an improvement in cognitive abilities assessed by Raven’s Progressive Matrices. However, we should point out that at baseline, they achieved high scores (average = 87th percentile for all children). A possible explanation for our results concerning the lack of significant differences between groups might be related to findings from previous studies, which suggest that individuals with lower starting cognitive performance have greater potential for improvement [66,100]. In contrast, those with higher initial performance have fewer opportunities for further optimization due to a ceiling effect. This means that children who may already be performing at a high cognitive level, like in our study, might show relatively smaller improvements as they are closer to their cognitive potential, leaving less room for significant gains.

## 5. Conclusions

The results of this research extend the discussion on the importance of the type and intensity of physical activity for the development of cognitive functions in children. While some systematic reviews underscore those dynamic exercises, i.e., aerobic training that facilitates executive functions [42,91,101], other studies favor activities that combine physical and cognitive effort [71,77,86]. However, there are some noted discrepancies regarding how physical activity impacts specific executive functions [35,71,102].

Different researchers try to disentangle the factors contributing to discrepancies in research findings related to exercise and executive functions, such as intensity, length, or type of exercise [42,71,103]. For example, exergames, dancing, and jogging can produce different cognitive demands and outcomes. Some activities might require more coordination or engagement than others, leading to varied cognitive benefits; the cognitive load of the exercise: activities with higher cognitive demands (e.g., sports requiring strategy or multitasking) may stimulate executive functions differently than simpler, repetitive exercises. Finally, specific tests used to assess executive functions (e.g., Stroop Test, Flankier task, and the Go/No-Go Task) can vary in sensitivity to changes in cognitive performance, leading to differing results. Different executive functions, such as working memory, inhibitory control, and cognitive flexibility, may be impacted in distinct ways based on the nature of the exercise. Thus, these variations can contribute to inconsistencies in the findings of studies on the effects of exercise on executive functions.

To conclude, our results suggest that a combination of both physical exercise and cognitive games on the Interactive Floor resulted in greater improvement in cognitive abilities in children than aerobic exercise or physical education lessons. There is agreement [104,105,106] that educational games designed with a “moving with thought” approach are particularly effective in promoting both physical health and cognitive development in children. These games not only increase energy expenditure but also immerse children in cognitively challenging situations. By combining physical activity with cognitive engagement, this dual-focus strategy supports the development of key cognitive skills while promoting physical well-being. This holistic approach may offer a powerful tool for enhancing overall child development, benefiting both their physical health and academic performance [77].

## Figures and Tables

**Table 1:** Examples of games from the Interactive Floor training.

Name of the Game	Description	Screen Shot
“warm up” set		
Fishes	Sensory app with interactive water that makes natural sounds when stimulated and swimming fish that respond to touch. The app has 5 different background images the instructor can change with the remote control. Stimulation with a hand, foot, other body part, or real object causes splashes and waves to spread on the water.	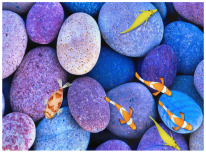
Bursting balloons	A relaxing game that trains coordination, agility, and reaction speed. It involves smashing escaping balloons by shading them (with a selected part of the body—hand or leg). The players collect points visible in the corner of the screen.	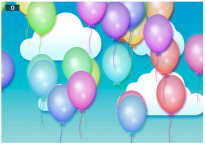
Happy Faces	Different emotions appear and disappear. The game is played by jumping on the appearing happy face, which will thus stay on the screen.	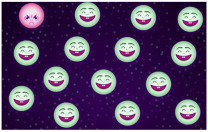
“we learn” set		
Pipeline	In the first level, the player’s goal is to rotate pieces of pipes using movement so that water can flow through them. At the learning level, there is a big task to code the path of the water by planning the rotation of the different pieces and creating a code in which the position is indicated by a letter and a number and the rotation of the tile by the letter “L” and “P.” Only after writing all the code will we see the result.	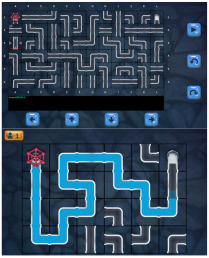
Encoding-Decoding	It is a 3-level math and logic game. Learn to work better in a team and learn the basics of programming without using a computer. It teaches detecting similarities and differences and exercises players’ perceptiveness.	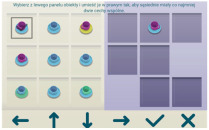
Seaport	Seaport is a game with multiple levels of difficulty. Its goal is to control the loading robot to deliver the crates to the designated places. Some of them have an additional time constraint, requiring even more planning of their movements. It develops teamwork skills using technology, programming, and robots.	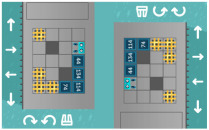
“we play” set		
Sea battle	A game for cooperation, perceptiveness, and speed of reaction involving fishing trash out of the water while sitting on a raft. Depending on the level, some trash is hard to pull out of the water and requires very intense waving over it, and some of it will also be hidden under aquatic vegetation. As the game advances and you progress, the water changes color to become more and more clear.	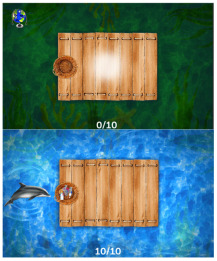
Chameleons	A dexterity, coordination-developing game for four people in which the goal is to collect 10 points as quickly as possible by hunting 10 small flies (waiting for the right moment to turn the chameleon’s head). Catching a large beetle deducts points	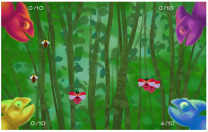
Rockets	The rocket game involves jumping on any flying rocket to prevent it from reaching Mars. It is a group game that teaches agility, cooperation, and coordination. The time to complete the task is limited.	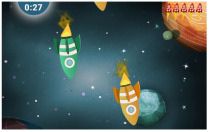

**Table 2:** Description of the tests used in the study.

Test	Description	Measurement
The Stroop Test assesses cognitive processing and response inhibition by evaluating how individuals process conflicting information.	The test is based on the concept that it takes longer to name the color of a word when the color does not match the word’s meaning (Interference tendency), e.g., the word “red” written in blue ink. It is calculated according to baseline conditions where a subject reads color words or names the color of the font itself.	For statistical analysis, two conditions are calculated. Reading Interference tendency (RIT): The difference in the reaction time (RT) medians between the reading speed in the color interference condition and the color-word reading baseline. Naming Interference tendency (NIT): The difference in the reaction time (RT) medians between naming speed in the word interference condition and the baseline color-naming speed.
The Corsi Block Test assesses visuospatial working memory.	In this test, a series of blocks (from 1 to 5) light up in a specific sequence, and the subject’s task is to replicate this sequence. First, the subject must replicate the sequence in the same order as presented (forward span); next the subject must replicate the sequence in reverse order (backward span).	The Corsi Block index score is calculated as the sum of points from both the forward and backward conditions, providing a composite measure of working memory span.
The Digit Span subtest assesses auditory working memory.	There are two tasks. In first the child listens to a sequence of numbers and must repeat them in the exact order (Digit Span Forward); in the second the child listens to a sequence and must repeat it in reverse order (Digit Span Backward).	Each item contains two trials, with each trial scored as either 1 (correct) or 0 (incorrect). The total score across both tasks forms the Digit Span index, which is used for analysis.
Raven’s Progressive Matrices assess fluid intelligence and non-verbal abstract reasoning.	The test consists of 60 visual matrices divided into five sets (A to E), each containing 12 items progressively increasing in difficulty. In each matrix, the subject must select the correct piece from several options to complete a pattern.	The final score is the total number of correct answers, indicating the individual’s abstract reasoning and problem-solving skills. This raw score was converted into a percentile rank (Raven’s Percentile) for statistical analysis.

**Table 3:** Means (M) and standard deviations (SD) for children’s scores in each Stroop Condition before (pre-test) and after intervention (post-test), separate for the 3 intervention groups. *p* score for the significant effects.

	Stroop Conditions								
Variable	Reading Interference Tendency (RIT)				Naming Interference Tendency (NIT)				*p*
**Time**	**Pre-Test**		**Post-Test**		**Pre-Test**		**Post-Test**		Time *p* < 0.001 Time × Group *p* = 0.028
**Group**	**M**	**SD**	**M**	**SD**	**M**	**SD**	**M**	**SD**	
Interactive Floor (IF)	0.160	0.116	0.031 *	0.004	0.127	0.149	0.027 *	0.006	
Aerobic Training (AT)	0.156	0.129	0.074 *	0.069	0.079	0.075	0.075	0.072	
Control (CON)	0.120	0.106	0.087	0.068	0.113	0.109	0.068 *	0.081	

Note: The symbol * presented in the post-test columns indicates significant differences between pre- and post-test results as measured by *t*-tests with Bonferroni correction for multiple comparisons.

**Table 4:** Means (M) and standard deviations (SD) for children’s scores in the Corsi Block Index before (pre-test) and after intervention (post-test), separate for the 3 intervention groups. *p* score for the significant effects.

Variable	Corsi Block Index				
Time	Pre-Test		Post-Test		*p*
**Group**	**M**	**SD**	**M**	**SD**	Group *p* = 0.004 Time *p* < 0.001 Time × Group *p* < 0.001
Interactive Floor (IF)	6.95	1.46	9.82 *	1.00	
Aerobic Training (AT)	7.29	0.85	8.48 *	1.36	
Control (CON)	7.09	1.09	7.85 *	0.96	

Note: The symbol * presented in the post-test columns indicates significant differences between pre- and post-test results as measured by *t*-tests with Bonferroni correction for multiple comparisons.

**Table 5:** Means (M) and standard deviations (SD) for children’s scores in the Corsi Block index before (pre-test) and after intervention (post-test), separate for the 3 intervention groups. *p* score for the significant effects.

Variable	Digital Span Index				
Time	Pre-Test		Post-Test		*p*
**Group**	**M**	**SD**	**M**	**SD**	Time *p* < 0.001
Interactive Floor (IF)	14.68	3.60	15.73	3.50	
Aerobic Training (AT)	15.28	2.87	16.10	2.23	
Control (CON)	14.62	2.42	15.43	2.09	

**Table 6:** Means (M) and standard deviations (SD) for children’s Raven’s percentile scores before (pre-test) and after intervention (post-test), separate for the 3 intervention groups. *p* score for the significant effects.

Variable	Raven’s Percentile				
Time	Pre-Test		Post-Test		*p*
**Group**	**M**	**SD**	**M**	**SD**	Time *p* < 0.001
Interactive Floor (IF)	84.82	18.77	95.05 *	6.33	
Aerobic Training (AT)	86.43	11.42	95.14 *	3.86	
Control (CON)	89.95	12.75	94.33 *	9.66	

Note: The symbol * presented in the post-test columns indicates significant differences between pre- and post-test results as measured by *t*-tests with Bonferroni correction for multiple comparisons.

## Data Availability

The original contributions presented in this study are included in the article. Further inquiries can be directed to the corresponding author.
